# Heterogeneity of circulating epithelial tumour cells from individual patients with respect to expression profiles and clonal growth (sphere formation) in breast cancer

**DOI:** 10.3332/ecancer.2013.343

**Published:** 2013-08-23

**Authors:** M Pizon, D Zimon, S Carl, U Pachmann, K Pachmann, O Camara

**Affiliations:** 1 Transfusion Center Bayreuth D-95448, Germany; 2 Universitätsklinikum der Friedrich Schiller Universität Jena D-07747, Germany; 3 Hufeland-Klinik Bad Langensalza D-99947, Germany

**Keywords:** circulating epithelial tumour cells, tumour spheres, breast cancer

## Abstract

**Background:**

The detection of tumour cells circulating in the peripheral blood of patients with breast cancer is a sign that cells have been able to leave the primary tumour and survive in the circulation. However, in order to form metastases, they require additional properties such as the ability to adhere, self-renew, and grow. Here we present data that a variable fraction among the circulating tumour cells detected by the Maintrac^® ^approach expresses mRNA of the stem cell gene NANOG and of the adhesion molecule vimentin and is capable of forming tumour spheres, a property ascribed to tumour-initiating cells (TICs).

**Patients and methods:**

Between ten and 50 circulating epithelial antigen-positive cells detected by the Maintrac approach were selected randomly from each of 20 patients with breast cancer before and after surgery and were isolated using automated capillary aspiration and deposited individually onto slides for expression profiling. In addition, the circulating tumour cells were cultured without isolation among the white blood cells from 39 patients with breast cancer in different stages of disease using culture methods favouring growth of epithelial cells.

**Results:**

Although no epithelial cell adhesion molecule (EpCAM)-positive cells expressing stem cell genes or the adhesion molecule vimentin was detected before surgery, 10%–20% of the cells were found to be positive for mRNA of these genes after surgery.

Tumour spheres from circulating cells of 39 patients with different stages of breast cancer were grown without previous isolation in a fraction increasing with the aggressivity of the tumour.

**Summary:**

Here we show that among the peripherally circulating tumour cells, a variable fraction is able to express stem cell and adhesion properties and can be grown into tumour spheres, a property ascribed to cells capable of initiating tumours and metastases.

## Background/Introduction

The incidence of breast cancer is increasing in the western world, in part due to demographic development and possibly due to lifestyle factors. Diagnosis raises fear and agitation in the affected patients. In spite of great efforts to develop methods for early detection and screening programmes, improved surgical approaches, and advanced therapies, success in preventing metastasis formation is limited, and the costs of some treatments are on the verge of becoming unaffordable within the scope of our health system.

Initial therapies aim at eliminating the main cancer burden by removal of the primary tumour. However, the most life-threatening aspect is not its growth but rather the capacity of the primary tumour to release cells into the blood circulation, which then can settle in vital organs and grow into metastases, impairing the function of these organs and eventually leading to fatal outcomes. Even if the tumour has been completely removed, many patients will later develop metastases, sometimes even after decades. Thus, in 90% of breast, prostate, and colon cancer patients, no metastases are detectable at the time of diagnosis, but even after complete removal of the primary tumour, more than 25% of these patients will develop fatal metastases during the further course of disease. Therefore, some cells must be able to survive over prolonged periods [1] and regrow in distant organs [2]. It has been proposed that neoplasms may be regarded as stem cell systems in which a minority of cells have the proliferative capacity to maintain the tumour, whereas the majority of cells demonstrate differentiation features and have limited proliferative potential [3]. The so-called cancer stem cells seem to be a subpopulation of tumour cells that possess the stem cell properties of self-renewal, differentiation, and generating the heterogeneous lineages of cancer cells [4]. A functional approach, ‘non-adherent sphere culture,’ is widely used to enrich the potential cancer stem cell subpopulations *in vitro*. Cells with stem cell-like properties are assumed to be the ones capable of growing into metastases [5].

There is still no agreement on how many cells are released from the primary tumour and can remain in the circulation [6–9]. It has been claimed that circulating epithelial cells suspected to be of tumour origin can be detected in the peripheral blood of tumour patients only after enrichment; however, even rare blood cell counting methods avoid manipulation of the samples for correct enumeration [10]. A method developed by us using a non-dissipative approach in analogy to other blood cell counting methods [11] allows us to reproducibly enumerate the cells suspected to be of tumour origin at different times during the course of disease and follow their behaviour and the development of their numbers over time.

Certainly not all tumour-derived cells detected in blood are capable of forming metastases [12], but such cells must be among those that have dissociated from the tumour and circulate in peripheral blood. Already more than ten years ago, it was shown that there must be cells in blood that can form xenografts [13], indicating that these cells can develop into metastases in patients. It is not known to which extent and at what time such cells can dissociate from the primary tumour and enter the blood stream [14], but it is assumed that this can occur from the moment of vascularisation [15]. How and when these cells tend to resettle may differ among various types of tumours [16] and even among tumours of the same type, depending on the patient’s condition [17] and the treatment provided.

The prerequisites for tumour cells to be able to form distant metastases are as follows:

1. They must be able to detach from the primary tumour.
2. They must be able to survive in the blood stream.
3. They must be able to re-adhere.
4. They must be able to initiate clonal growth at the distant site.
5. They must be able to initiate or attract vessel formation for the supply of nutrients in order to grow into life-threatening metastases.

If circulating tumour cells are detectable, it proves that these cells have accomplished the first two steps of detaching from the tumour and surviving in the circulation. Cells that have accomplished the third step must have travelled via the blood to distant sites; however, they will no longer be detectable in the peripheral blood. It would be a fundamental step ahead to be able to identify the cell type among the circulating tumour cells that is capable of performing the three last steps and is therefore responsible for metastatic growth. It would provide valuable insight into whether or not such cells are actually present in the patient, how fast they can duplicate [18], how they respond to therapy, whether and how their properties change during the course of the disease [19, 4], and how long they can survive in the patient [17].

In this article, we show that cells detected by our non-dissipative approach can be randomly isolated individually. They can be analysed for their heterogeneity in expression profiles of stem cell and adhesion markers and for changes of these properties before and after surgery. In addition, the self-renewal ability of tumour cells circulating in the blood can be analysed for the first time, showing that a fraction of disseminated cells detected by our Maintrac approach can be induced to grow and form spheres of cells [20–22] and thus have the properties of tumour and metastasis-initiating cells.

## Patients and Methods

Circulating tumour cells from 20 patients with newly diagnosed primary breast cancer were subject to expression analysis before and between two and three weeks after surgery. For detection of circulating tumour cells with the ability for clonal growth as tumour spheres, cells from the blood of 39 breast cancer patients in different stages of disease (Table 1) and ten healthy subjects were cultured for up to 28 days.

Blood samples (2–7 mL) were drawn into standard blood count tubes, containing ethylenediaminetetraacetic acid (EDTA) as an anticoagulant. The enumeration of circulating epithelial tumour cells (CETCs) was performed using a non-dissipative Maintrac approach, as reported previously [11]. In short, in order to compensate for shipping delays, 1 mL samples were subjected to red blood cell lysis at day two after blood sample collection (with usually 95% viability), using 10 mL of erythrocyte lysis solution (Qiagen Hilden, Germany) for 10 min at room temperature, spun down at 700×g and re-diluted in 1 mL of PBS (phosphate-buffered saline) pH 7.4. Ten microlitres of fluorescein isothiocyanate (FITC)-conjugated mouse antihuman epithelial antibody (HEA, Miltenyi, Bergisch Gladbach, Germany) was added to 100 μL of cell suspension incubated for 15 min in the dark, readjusted with PBS to 1 mL, and a defined volume of the cell suspension was applied to a defined area into wells of an enzyme linked immunosorbent assay (ELISA) plate. The cells were measured using image analysis in a Scan-R microscope (Olympus, Munich, Germany). The values were displayed in scattergrams and histograms. This enables the user to locate cells contained within the desired population for visual examination and to take fluoromicrographs [11].

**Figure 1:**
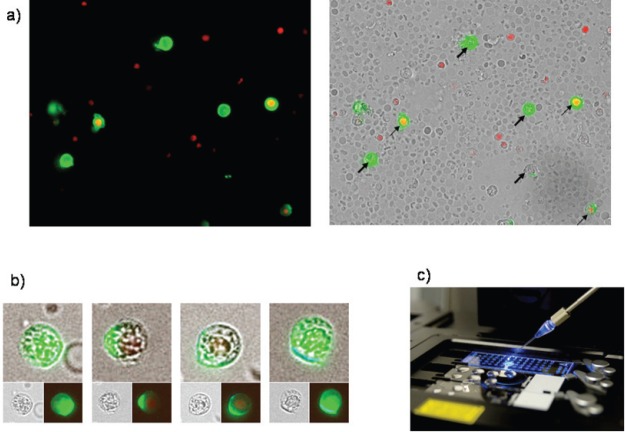
(a) Four vital epithelial antigen-positive tumour cells (only green fluorescent, thick arrows) and three avital (green and red fluorescent, thin arrows) among the unstained live and red fluorescent nuclei of avital blood cells. (b) Typical cells used for individual selection in transmitted light and green fluorescence (small pictures) and the merged pictures (large pictures). (c) The semiautomated capillary device for aspiration of individual cells from cell suspensions (slides in the front) and deposition in indiviual wells (slide in the background).

For expression analysis, vital cells stained with membrane epithelial cell adhesion molecule (EpCAM) and excluding propidium iodide (PI) as an avitality marker among the white blood cells from 20 breast cancer patients before and after surgery, comprising the tumour suspect cells from 1 mL of blood, were selected individually using the CellEctor Plus (MMI, Zurich, Switzerland) and deposited one by one onto Advalytix AmpliGrid Microscope Slides (Hamilton) (Figures 1a–c).

### Amplification of cDNA from CETCs

cDNA amplification from a single CETC was performed using a Takara CellAmp™ Whole Transcriptome Amplification Kit (Takara Bio Inc.) according to the manufacturer’s directions. Briefly, for cell lysis, 1 μL lysis buffer was added directly to the isolated single cell under an oil cover. Slides were placed in an Advalytix (Beckmann, Germany) thermocycler and heated to 72°C for 1 min, followed by 5 min at 4°C. After cell lysis, 1 μL of RT-enzyme mix was added to the lysed single cell. The reverse transcription was performed at 42°C for 5 min, followed by a 5-s 85°C inactivation step. Subsequently, 0.5 μL enzyme mix was added to each cell on the slide and poly (A) addition reaction was performed at 37°C for 15 min, followed by 10 min at 70°C. After cDNA synthesis and poly (A) addition reaction, 2.5 μL of polymerase chain reaction (PCR) reaction mixture was added directly to the unpurified products, followed by amplification using a PCR cycling profile, which consisted of one cycle of 95°C for 3 min, 50°C for 2 min, and 72°C for 3 min; 20 cycles of 95°C for 30 s, 67°C for 1 min, and 72°C for 3 min; and one cycle of 72°C for 10 min. The primer sequences and length products of each gene are shown in Table 2. Finally, PCR products were electrophoresed on a 2% agarose gel and stained with ethidium bromide.

Patients with more than 100 CETCs per 100 μL of cell suspension were enrolled for a further culture of tumour spheres. After erythrocyte lysis and one centrifugation step, all white blood cells from 1 mL of blood were plated in 7.5-mL (25-cm^2^) culture flasks at a density of 10^3 ^cells/mL in RPMI-1640, supplemented with low concentration of fetal bovine serum and L-glutamine and the epidermal growth factor (EGF), insulin, and hydrocortisone. Cells were incubated for 28 days with the addition of fresh medium every five days at 37°C in 5% humidified CO_2_. The formation of tumour spheres was observed every seven days under an inverted light microscope (Primo Vert Zeiss, Germany) at 40× magnification. After the seventh, 14th, and 21st day of cultures, the cultures containing the tumour spheres were collected and centrifuged at 780×g for 7 min. The supernatant was discarded and the pellet resuspended with 50 μL PBS, and the cell suspension was transferred into 1.5 mL reaction tubes, stained with 5 μL of FITC-conjugated EpCAM (Miltenyi Biotec GmbH, Germany) and incubated for 15 min in the cold. The samples were subsequently diluted with 430 μL PBS-EDTA and then stored overnight at 4°C. One hundred microlitres of cell suspension and 5 μL of PI (Sigma-Aldrich, USA) were transferred to the wells of ELISA plates (Greiner Bio-one, USA). Analysis of the red and green fluorescence of the tumour spheres was performed using a fluorescence scanning microscope (ScanR Olympus, USA). Vital tumour spheres were defined as EpCAM positive, lacking in PI staining, and with intact morphology. Finally, only vital tumour spheres were counted, and the results were calculated as spheres/mL blood.

## Results 

**Figure 2:**
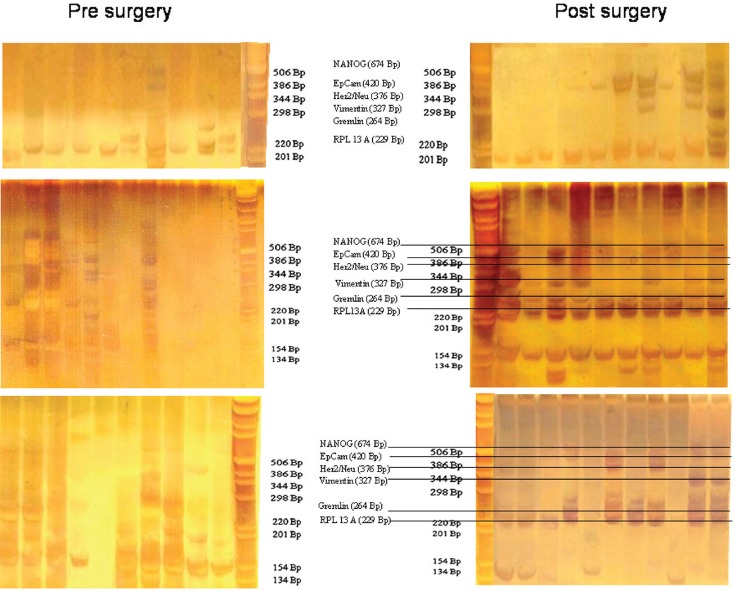
Gel analysis of expression profiles of 10–12 cells from three patients isolated before and after surgery. Each lane is the profile derived from a single cell.

For expression analysis, all white blood cells comprising the tumour suspect cells from 1 mL of blood were stained with green fluorescence labelled anti-EpCAM. Vital epithelial cells (10–12) from 20 breast cancer patients before and after surgery stained with membrane EpCAM and excluding PI as an avitality marker were selected individually, deposited one by one, and analysed for the mRNA expression of EpCAM, human epidermal growth factor receptor 2 (HER2)/neu, vimentin, and NANOG (homeobox protein), and the two housekeeping genes Gremlin and RPL 13 A. In 20/20 cases, cells showed increased expression after surgery as compared to before surgery. Three typical comparisons are shown in Figures 2a–c. In a patient at the end of preoperative chemotherapy, expression of at least one housekeeping gene was seen in all of the ten surface EpCAM-positive cells randomly picked before surgery. However, clear mRNA expression of EpCAM was detected only in one cell in which expression of the stem cell gene NANOG was also seen. After surgery, in contrast, EpCAM mRNA was detected in four out of ten cells, HER2/neu expression in six out of ten cells, vimentin (an adhesion molecule, which is characteristic for epithelial-mesenchymal transition) in three out of ten cells, and NANOG in one out of ten cells, indicating that the cells present after surgery showed a higher activation status of genes relevant for growth activity and adhesion than those before surgery. This was also true for the expression of HER2/ neu, although the primary tumour had been classified as HER2/neu-negative. The increase in gene expression was even more obvious in the second patient, in whom prior to surgery for invasive ductal carcinoma (pT1c N1a (1/49) L1 V0 G3 R0, M0 ER: 90%, PR: 20%, HER-2/ neu-negative) all of the 12 randomly picked surface EpCAM-positive cells tested showed no relevant expression of any of the genes. After surgery, circulating numbers of tumour suspect cells had increased by fourfold, and a clear expression of both tested housekeeping genes was seen in ten out of ten cells selected. EpCAM mRNA was now clearly detected in six out of ten cells, HER2/neu in eight out of ten cells, vimentin in six out of ten cells, and NANOG in three out of ten cells. EpCAM and vimentin were coexpressed in three out of ten cells, EpCAM and HER2/neu in five out of ten cells, NANOG and HER2/neu in two out of ten cells, and vimentin and NANOG in two out of ten cells.

Comparable effects were seen in a third patient with a ductal invasive tumour (pT1c L1 V1 pN0 (sn), G3, R0 ER-, PR-, HER-2/neu-negative) and thus, too, triple negative. Hardly any mRNA expression was seen in all ten surface EpCAM-positive cells prior to surgery. After surgery, 11 cells could be analysed. Nine out of 11 surface EpCAM-positive cells showed expression of the housekeeping genes, five out of 11 showed expression of EpCAM mRNA, two out of 11 showed expression of HER2/neu, two out of 11 showed expression of vimentin, and three out of showed 11 expression of NANOG. EpCAM and vimentin were coexpressed in two out of 11 cells, EpCAM and HER2/neu in two out of 11 cells, EpCAM and NANOG in three out of 11 cells, and vimentin and NANOG in two out of 11 cells. Thus, surgery led to the activation of genes considered necessary for adhesion and stem cell activity in about 30% of cells of epithelial origin in the peripheral blood of breast cancer patients. This activation may enable such cells to leave the circulation early after surgery and become adherent in foreign tissue. But only if they are then able to grow can they form metastases and contribute to early relapses, which peak two to three years after surgery. The expression status of the tumour cells remaining in the circulation may subsequently be modified by ensuing chemotherapy, radiotherapy, and/or hormone-blocking therapy.

The ultimate proof, however, that epithelial antigen-positive cells suspected to have tumour affiliation present in peripheral blood can be the origin of metastases is their ability to grow clonally and form spheroid cell clusters.

**Figure 3:**
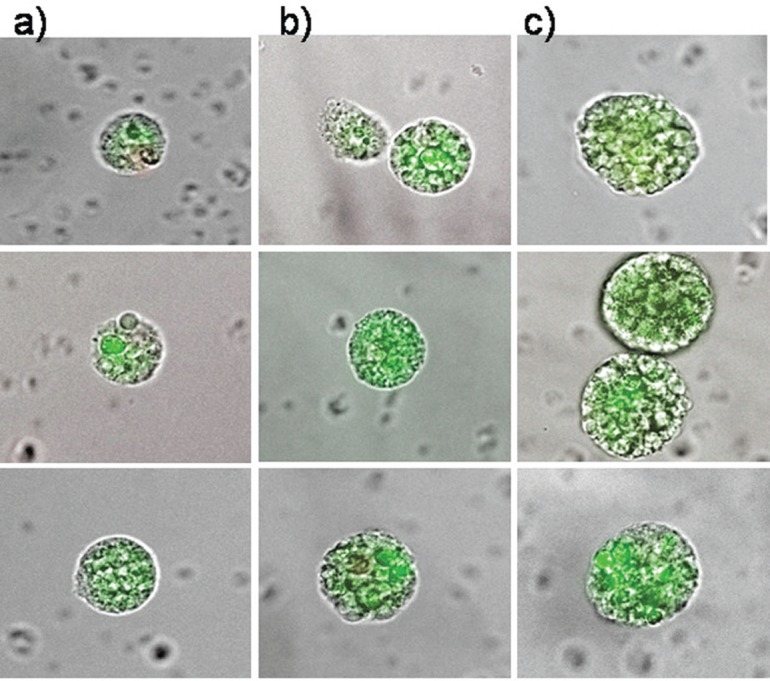
The typical clonal expansion of cells (tumour spheres) originating from circulating epithelial cells from peripheral blood of three breast cancer patients stained with FITC-anti-EpCAM at (a) day 7, (b) day 14, and (c) day 21. The pictures are a merge of transmitted and fluorescent light.

Therefore, circulating epithelial antigen-positive tumour suspect cells were analysed for cells capable of forming cell spheres under culture conditions favouring the growth of epithelial cells. For the present analysis, 39 patients in different stages during the course of disease were chosen (Table 1) in whom the range of CETC detectable using the non-dissipative Maintrac approach ranged from 1,700 to 9,360 cells/mL (equivalent to 0.2–1.3‰ of white blood cells). One millilitre of blood from each patient was prepared as described in the “Patients and Methods” section and cultured under non-adherent suspension conditions for up to 28 days. Spheroid cell clones developed in 79% of the patients. Typical spheres, increasing in size over time from 7–21 days, are shown in Figures 3a–c, demonstrating that clonal growth in a fraction of the tumour suspect cells present in the blood persisted for at least three weeks.

**Figure 4:**
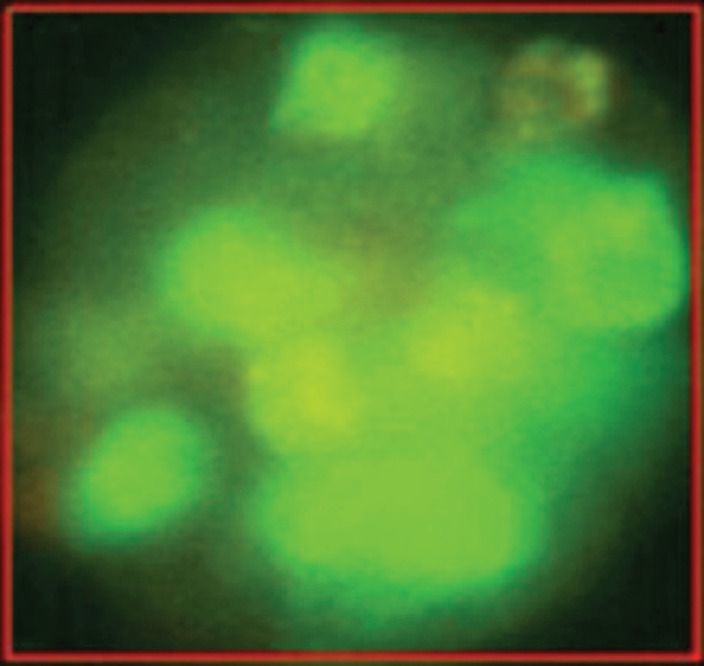
The variability of EpCAM expression in cells from a sphere derived from one single cell as shown in fluorescent light.

The epithelial nature of the spheres was determined by staining with a fluorochrome-labelled anti-EpCAM antibody. Staining with anti-EpCAM was variable among the cells from the same sphere, as evidenced by the inspection of the spheres in fluorescent and transmitted light (Figures 3 and 4). Thus, it is obvious that EpCAM expression can already differ in the progeny from one TIC. No spheres without EpCAM staining were observed, confirming the epithelial nature of the spheres. No sphere formation was observed in blood cells from ten healthy subjects.

**Figure 5:**
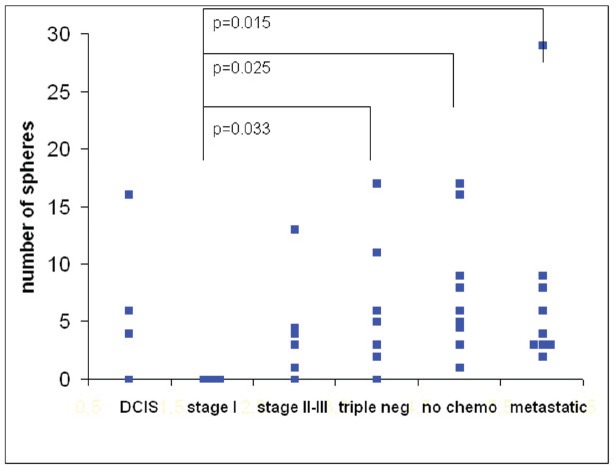
The number of tumour spheres per millilitre of blood from 39 breast cancer patients in different stages of disease at 21 days of culture. The differences were significant between patients with stage I tumours versus triple-negative tumours (*p* = 0.033), stage II–III tumours prior to chemotherapy (*p*= 0.025), and metastatic disease (*p*= 0.015).

The number of spheres was determined from 1 mL of blood from 39 breast cancer patients. The numbers of spheres varied from 0–29/mL. The distribution of sphere numbers from patients with breast cancer at different stages is shown in Figure 5. Surprisingly, the number of sphere-forming cells was widely distributed in patients with ductal carcinoma *in situ *(DCIS), whereas sphere-forming cells were absent in all three patients with stage I tumours. Patients with triple-negative breast cancer and patients with metastatic disease exhibited significantly higher levels of sphere-forming cells than patients with stage I tumours (*p *= 0.033 and 0.015, respectively) and somewhat higher levels than patients with stage II–III tumours treated according to the guidelines. Most importantly, patients with non-metastatic tumours who had been operated on and not yet received any chemotherapy exhibited almost the same high numbers of sphere-forming cells (*p *= 0.025) as those found in patients with metastatic disease.

Thus, our first results indicate that the ability of a subpopulation of circulating tumour cells to grow into spheres correlates with the aggressiveness of the tumour.

## Discussion

The most frightening aspect of solid tumours is their ability to develop metastases in distant organs, eventually leading to fatal organ failure. To prevent such dissemination, surgeons performed radical mastectomies, based on the assumption that it was necessary to remove as much tissue as possible to prevent further spread of the cancer. Fisher [23] challenged this hypothesis claiming that breast cancer was potentially a systemic disease with tumour cells infiltrating other organs of the body. Subsequent adjuvant chemotherapy to eradicate residual systemic disease [24] showed that chemotherapy and/or hormonal therapy is effective in the management of women with estrogen-receptor (ER)-negative or ER-positive tumours. But in spite of adjuvant chemotherapy, a considerable percentage of patients still suffer from relapses. It was hypothesised that this is due to the resistance of occult micrometastases to chemotherapy, and in the adjuvant situation, there is, so far, no tool available to determine which tumours are resistant to therapy.

Preoperative chemotherapy expected, which reduces or eliminates occult micrometastases [25] and provides a possibility to test the sensitivity of the tumour to the drugs, failed to show that it was non-inferior to adjuvant chemotherapy [26], indicating a lack of influence on preoperative micrometastatic disease. The prognostic relevance of tumour response showed a paradoxic outcome [27], with the poorest outcome in highly chemosensitive tumours—such as triple negative tumours—challenging the impact of preoperative micrometastases on tumour relapse. This is corroborated by the observation that tumour cells detected in bone marrow are not necessarily an indication of relapse [28].

On the contrary, cells disseminated during neoadjuvant chemotherapy [29] and surgery [30] might be activated and capable of regrowing into metastases, especially in patients with triple-negative tumours [31] who do not receive any additional maintenance therapy, such as hormone-blocking therapy or immunotherapy with trastuzumab, which might be able to silence such cells.

In this article, we investigate the effect of surgery on the peripherally circulating tumour cells, using an approach where peripheral blood cells from patients with malignant epithelial tumours are subject to minimal perturbation with only the red blood cells being removed by lysis. The range of tumour suspect cells detected is between 1/100,000 and 1/1,000 in primary tumours. This is more than the number reported using magnetic bead enrichment methods that require a certain level of surface antigen expression for the cells to be retained in the magnetic field and thus are prone to selection bias [32].

Taking all cells, even those with minimal visually detectable EpCAM expression, yielded sufficient cells to allow for random selection of cells for single cell isolation. With respect to EpCAM surface antigen of the isolated cells, there was no difference between cells before and after surgery; in both cases, the expression profiles differed significantly, and this was true for all 20 patients. In neoadjuvantly treated patients, typically more cells isolated after surgery showed high transcriptional activity as compared to before surgery. This phenomenon was even more prominent in patients without treatment before surgery in whom most of the cells isolated before surgery showed very low transcriptional activity, although, according to our criteria, they were viable cells. In contrast, a high transcriptional activation status of two housekeeping genes was seen in all of the 10–20 single cells that were randomly selected after surgery. This is a proof that these cells are viable. The expression profile of the four other genes showed a high transcriptional heterogeneity in these cells. After surgery, EpCAM was clearly expressed in most of the cells. In addition, in some of the cells, HER2/neu and the stem cell-associated NANOG gene were activated. Recent research has related cancer stem cells to a regulatory cellular process known as the epithelial–mesenchymal transition, during which epithelial cells acquire the ability to invade, resist apoptosis, and disseminate [2]. Vimentin, a protein expressed during epithelial–mesenchymal transition, was found in a considerable percentage of cells after surgery, sometimes together with NANOG. Thus, we observed the expression of adhesion and stem cell genes in part of the presumably previously dormant cells, which is postulated to be a prerequisite for metastasis formation. Most importantly, the transcriptional activity was shown to change in a treatment-dependent manner during the course of the disease, indicating that the phenotype of markers proposed in the literature to identify cancer stem cells might also be dynamically switched. Our results indicate that surgery can induce cells to undergo such a transition with coexpression of epithelial and mesenchymal mRNA and thus may contribute to early relapses [33].

In addition, the results of our study provide evidence indicating that expression profiles may not be stable traits of circulating tumour cells. Therefore, it is not surprising that in a recent report comparing the expression profiles of magnetic bead isolated cells from different patients and isolated cell line cells [34], the expression profiles of the circulating tumour cells did not cluster by patient or disease stage.

Even if expression profiles are relevant during the metastatic process, they may not be suitable for the characterisation of the metastatic potential of circulating tumour cells. Moreover, not all circulating tumour cells may be capable of growing into metastasis.

After adhesion, a further indispensable prerequisite for metastatic growth is the ability of the cells to undergo clonal expansion. Cells capable of growing clonally, called TICs, have been reported to form spheroid clusters, also termed tumour spheres.

If it were possible to identify the cells among the tumour cells in the blood that are capable of forming metastases, they could be used to estimate the risk of metastatic relapse and serve as a diagnostic tool for patient stratification, early determination of the therapy failure, or to determine the potential risk of resistance to the given therapeutic intervention [35]. More importantly, these cells could be addressed directly by further treatment options.

In this article, we show that such spheres can, with appropriate stimulation, be induced from peripherally CETCs. The epithelial nature of the spheres was unequivocally proven by staining for the epithelial marker EpCAM. Sphere formation has so far only been detected in blood from tumour patients. Moreover, the frequency of sphere forming cells correlated with the aggressiveness of the tumour, from stage I tumours lacking sphere-forming cells to triple-negative tumours with a frequency of sphere-forming cells comparable with tumours that had already formed metastases. Strikingly, patients who had only been operated on and had not yet received systemic therapy exhibited the highest numbers of sphere-forming cells. This is in line with our observation that surgery can mobilise tumour cells into the circulation, and these data show that surgery can also contribute to the activation of these cells.

Here we demonstrate that it is possible to stimulate a subpopulation of circulating tumour cells to induce them to grow into clonal spheroid clusters directly from peripheral blood.

## Conclusion

With a highly conservative approach, using only one washing step and no enrichment, we can not only detect high numbers of circulating epithelial cells suspected to be of tumour origin in different stages of disease but it also enables us to maintain these cells in viable condition, allowing a fraction of these cells to divide and grow into tumour spheres, a prerequisite for metastasis formation. To our knowledge, this is the first report showing that direct induction of tumour spheres from tumour cells circulating in peripheral blood is possible *in vitro*.

Our preliminary results indicate that the property to grow into spheres correlates with the aggressiveness of the tumour, even if, in the patient, this may require the appropriate environment and proper stimuli. Such stimulation may be provided by inflammatory signals, associated with factors such as surgery.

## Conflict of interest

The corresponding author, Dr. Pachmann, holds a patent 7615358 protecting the Maintrac method outside of Europe and is a partner in the SIMFO GmbH, a company dedicated to developing new tests but which does not sell tests to patients. Tests are performed for physicians and patients on request as medically indicated, and tests are supervised by the transfusion-medical laboratory of Dr. Ulrich Pachmann.

## Figures and Tables

**Table 1.** Clinical parameters of the 39 patients.

Pat. No	Initial stage	Current status	T	N	M	HR-status	Her2/neu
1	II	met	1	1	0	+	−
2	II	CR	n.a.	1	0	+	n.a.
3	I	met	1	0	0	n.a.	n.a.
4	II	CR	1	1	0	−	−
5	I	no chemo	1	0	0	+	−
6	I	no chemo	1	0	0	+	−
7	I	CR	1	0	0	+	−
8	dcis	CR	Tis	0	0	+	−
9	I	met	1	0	0	+	−
10	III	CR	ypT2	3	0	−	−
11	I	CR	1	0	0	+	−
12	dcis	CR	Tis	n.a.	0	+	+
13	IV	met	2	0	1	+	n.a.
14	II	CR	2	1	0	−	−
15	II	met	1	1	0	−	−
16	I	no chemo	1	0	0	+	−
17	II	CR	2	0	0	+	−
18	I	no chemo	1	0	0	+	−
19	I	no chemo	1	0	0	+	−
20	II	met	2	n.a.	n.a.	+	−
21	II	CR	2	1	0	−.	−
22	dcis	CR	Tis	0	n.a.	+	−
23	II	CR	1	1	0	−	−
24	I	CR	1	0	0	n.a.	n.a.
25	III	CR	4	1	0	+	−
26	II	no chemo	2	0	0	n.a.	n.a.
27	II	met	1	1	0	+	−
28	II	met	2	0	0	+	−
29	II	CR after neoadjuvant (primary) systemic chemotherapy	n.a.	n.a.	n.a.	−	−
30	III	CR	2	3	n.a.	−	−
31	II	no chemo	2	1	0	+	−
32	I	CR	1	micr.	0	+	+
33	III	no chemo	2	3	n.a.	n.a.	n.a.
34	I	no chemo	1	0	0	+	−
35	III	CR	3	1	n.a.	n.a.	−
36	dcis	CR	Tis	n.a.	0	+	−
37	III	no chemo	2	3	n.a.	n.a.	n.a.
38	II	CR	n.a.	1	0	+	−
39	II	met	2	1	n.a.	+	n.a.

HR: hormone receptor; CR: complete remission; met: metastatic; no chemo: chemotherapy not yet received or refused. Received; n.a.: not available; micr: less than 2-mm invasion.

**Table 2.** Forward and reverse primer sequences for EpCAM, NANOG, vimentin, Her2, RPL13A, and Gremlin.

Name	Product length	Primer sequence (s-sense; a-antisense)
EpCAM	219 bp	s: GGG AAA TAG CAA ATG GAC ACA a: CGA TGG AGT CCA AGT TCT GG
NANOG	674 bp	s: GGA TCC AGC TTG TCC CCA AA a: TGC ACC AGG TCT GAG TGT TC
Vimentin	327 bp	s: GGC TCA GAT TCA GGA ACA GC a: GCT TCA ACG GCA AAG TTC TC
Her2	367 bp	s: CGA GAG GTG AGG GCA GTT AC a: AGC AGA GGT GGG TGT TAT GG
RPL13A	229 bp	s: AGC TCA TGA GGC TAC GGA AA a: CTT GCT CCC AGC TTC CTA TG
Gremlin	264 bp	s: AAC TTG GCC TAC TGG CAA TG a: TCT CGA GTT GCA AGG GTT CT a: TCT CGA GTT GCA AGG GTT CT
